# Excitation of rat sympathetic neurons via M_1_ muscarinic receptors independently of K_v_7 channels

**DOI:** 10.1007/s00424-014-1487-1

**Published:** 2014-03-26

**Authors:** Isabella Salzer, Hend Gafar, Viola Gindl, Peter Mahlknecht, Helmut Drobny, Stefan Boehm

**Affiliations:** Department of Neurophysiology and Neuropharmacology, Center for Physiology and Pharmacology, Medical University of Vienna, Waehringerstrasse 13a, 1090 Vienna, Austria

**Keywords:** M_1_ muscarinic receptors, Noradrenaline release, K_v_7 channels, Protein kinase C, Ca^2+^-activated Cl^−^ channels

## Abstract

The slow cholinergic transmission in autonomic ganglia is known to be mediated by an inhibition of K_v_7 channels via M_1_ muscarinic acetylcholine receptors. However, in the present experiments using primary cultures of rat superior cervical ganglion neurons, the extent of depolarisation caused by the M_1_ receptor agonist oxotremorine M did not correlate with the extent of K_v_7 channel inhibition in the very same neuron. This observation triggered a search for additional mechanisms. As the activation of M_1_ receptors leads to a boost in protein kinase C (PKC) activity in sympathetic neurons, various PKC enzymes were inhibited by different means. Interference with classical PKC isoforms led to reductions in depolarisations and in noradrenaline release elicited by oxotremorine M, but left the K_v_7 channel inhibition by the muscarinic agonist unchanged. M_1_ receptor-induced depolarisations were also altered when extra- or intracellular Cl^−^ concentrations were changed, as were depolarising responses to γ-aminobutyric acid. Depolarisations and noradrenaline release triggered by oxotremorine M were reduced by the non-selective Cl^−^ channel blockers 4-acetamido-4′-isothiocyanato-stilbene-2,2′-disulfonic acid and niflumic acid. Oxotremorine M induced slowly rising inward currents at negative membrane potentials that were blocked by inhibitors of Ca^2+^-activated Cl^−^ and TMEM16A channels and attenuated by PKC inhibitors. These channel blockers also reduced oxotremorine M-evoked noradrenaline release. Together, these results reveal that slow cholinergic excitation of sympathetic neurons involves the activation of classical PKCs and of Ca^2+^-activated Cl^−^ channels in addition to the well-known inhibition of K_v_7 channels.

## Introduction

Acetylcholine is the prime transmitter in the ganglia of the entire autonomic nervous system; it excites postganglionic neurons simultaneously via two different types of receptors: nicotinic (nAChRs) and muscarinic (mAChRs) acetylcholine receptors. Ganglionic transmission via these two receptors can occur independently of each other [8, 36]. However, the excitation of postganglionic neurons via mAChRs is much slower than that via nAChRs and involves a G protein-mediated inhibition of K_v_7 channels, also known as M-type K^+^ channels [9]. There are at least five different subtypes of mAChRs, named M_1_ through M_5_ [38], and the inhibition of K_v_7 channels in postganglionic sympathetic neurons was found to involve M_1_ receptors [5, 28]. The underlying signalling mechanism is a phospholipase C-mediated depletion of phosphatidylinositol-4,5-bisphosphate (PIP_2_) in the neuronal plasma membrane [11].

In primary cultures of postganglionic sympathetic neurons, activation of M_1_ mAChRs causes depolarisation and action potential firing, which ultimately leads to exocytotic noradrenaline release from the axon terminals [23, 27]. The following results indicated that an inhibition of K_v_7 channels contributed to this sequence of events: (1) retigabine, an activator of K_v_7 channels, abolished noradrenaline release evoked by the mAChR agonist oxotremorine M, but not that triggered by electrical field stimulation [23]; (2) direct inhibition of K_v_7 channels by Ba^2+^ and/or linopirdine also elicited action potential- and Ca^2+^-dependent noradrenaline release from sympathetic neurons [7, 20]; (3) activation of B_2_ bradykinin receptors on sympathetic neurons caused an inhibition of K_v_7 channels [18], on one hand, and led to noradrenaline release, on the other hand, again in an action potential- and Ca^2+^-dependent manner [7]. However, in the case of B_2_ bradykinin receptors, an additional mechanism was found to mediate sympathoexcitation caused by this peptide: an activation of protein kinase C [34]. In sympathetic neurons, protein kinase C (PKC) can also be activated via mAChRs [26], and PKC may also contribute to the muscarinic inhibition of K_v_7 channels [15], most probably by regulating the PIP_2_ sensitivity of the channel [24]. However, it is still unknown whether PKC might also be involved in the excitation of postganglionic sympathetic neurons via M_1_ receptors and, if so, whether a PKC-dependent excitation of sympathetic neurons also relies on an inhibition of K_v_7 channels.

Noradrenaline release from sympathetic neurons is not only triggered by an activation of M_1_ receptors, as described above, but is also modulated (i.e. enhanced or decreased) by several muscarinic receptors located at the axon terminals where exocytosis occurs. These presynaptic receptors generally mediate a reduction of action potential-evoked noradrenaline release, which is in most instances based on an inhibition of voltage-activated Ca^2+^ channels via pertussis toxin-sensitive G proteins [21]. Therefore, experiments regarding the release of noradrenaline were carried out on neurons treated with pertussis toxin in order to largely eliminate confounding effects of inhibitory presynaptic muscarinic receptors. The results demonstrate that the activation of M_1_ receptors can depolarise sympathetic neurons and induce noradrenaline release independently of K_v_7 channels; the alternative signalling mechanisms include classical PKC enzymes and Ca^2+^-activated Cl^−^ channels.

## Materials and methods

### Primary cultures of rat superior cervical ganglion neurons

Primary cultures of dissociated superior cervical ganglion (SCG) neurons from neonatal rats were prepared as described before [22]. Newborn Sprague–Dawley rats were kept and killed 3–10 days after birth by decapitation in full accordance with all rules of the Austrian animal protection law (see http://ris1.bka.gv.at/Appl/findbgbl.aspx?name=entwurf&format=pdf&docid=COO_2026_100_2_72288) and the Austrian animal experiment law (see http://www.ris.bka.gv.at/Dokumente/BgblAuth/BGBLA_2012_I_114/BGBLA_2012_I_114.pdf). The ganglia were removed immediately after decapitation of the animals, cut into three to four pieces and incubated in collagenase (1.5 mg ml^−1^; Sigma, Vienna, Austria) and dispase (3.0 mg ml^−1^; Boehringer Mannheim, Vienna, Austria) for 30 min at 36 °C. Subsequently, they were further incubated in trypsin (0.25 % trypsin; Worthington, Lakewood, NJ) for 15 min at 36 °C, dissociated by trituration and resuspended in Dulbecco’s modified Eagle’s Medium (InVitrogen, Lofer, Austria) containing 2.2 g l^−1^ glucose, 10 mg l^−1^ insulin, 25,000 IU l^−1^ penicillin and 25 mg l^−1^ streptomycin (InVitrogen), 50 μg l^−1^ nerve growth factor (R&D Systems Inc., Minneapolis, MN) and 5 % fetal calf serum (InVitrogen). Finally, all cells were seeded onto 5-mm plastic discs for radiotracer release experiments and onto 35-mm culture dishes for electrophysiological experiments. All tissue culture plastic was coated with rat tail collagen (Biomedical Technologies Inc., Stoughton, MA). The cultures were stored for 4–8 days in a humidified 5 % CO_2_ atmosphere at 36 °C. On days 1 and 4 after dissociation, the medium was exchanged entirely.

### Electrophysiology

Recordings were carried out at room temperature (20–24 °C) on the somata of single SCG neurons using the perforated-patch version of the patch-clamp technique which prevents the rundown of currents through K_v_7 channels [6]. Patch pipettes were pulled (Flaming-Brown Puller, Sutter Instruments, Novato, CA) from borosilicate glass capillaries (Science Products, Frankfurt/Main, Germany) and front-filled with a solution consisting of (in millimolars) K_2_SO_4_, 75; KCl, 55; MgCl_2_, 8; and HEPES, 10, adjusted to pH 7.3 with KOH. Then, electrodes were backfilled with the same solution containing 200 μg/ml amphotericin B or 50 μg/ml gramicidin D (in 0.8 % DMSO), which yielded tip resistances of 1–3 MΩ. In some current-clamp recordings, KCl in the pipette was replaced by identical concentrations of either CsCl or K-gluconate. For the measurement of oxotremorine M-induced currents, the pipette solution contained (in millimolars) KCl, 140; CaCl_2_, 1.0; MgCl_2_, 0.7; EGTA, 10; and HEPES, 10, adjusted to pH 7.3 with KOH. The bathing solution consisted of (in millimolars) NaCl, 140; KCl, 3.0; CaCl_2_, 2.5; MgCl_2_, 2.0; glucose, 20; and HEPES, 10, adjusted to pH 7.4 with NaOH. With this bath solution, liquid junction potentials ranged between −8 mV for pipette solutions containing K_2_SO_4_ plus KCl or CsCl and −12.4 mV for the pipette solution containing 55 mM K-gluconate. These values were corrected for during experimentation. Tetrodotoxin (0.5 μM) was included to suppress action potential firing which interferes with the precise determination of depolarisations. All other drugs were applied via a DAD-12 drug application device (Adams & List, Westbury, NY), which permits a complete exchange of solutions surrounding the cells under investigation within <100 ms. To investigate currents through K_v_7 channels, cells were held at a potential of −30 mV, and four times per minute 1-s hyperpolarisations to −55 mV were applied to deactivate the channels; the difference between current amplitudes 20 ms after the onset of hyperpolarisations and 20 ms prior to re-depolarisation was taken as a measure for currents through K_v_7 channels. Amplitudes obtained during the application of test drugs (*b*) were compared with those measured before (*a*) and after (*c*) application of these drugs by calculating 200b/(*a* + *c*) = % of control or 100 − (200*b*/[*a* + *c*]) = % inhibition [6].

### Determination of [^3^H]noradrenaline release

[^3^H]noradrenaline uptake and superfusion were performed as described [23]. Briefly, plastic discs with dissociated neurons were incubated at 36 °C for 1 h in 0.05 μM [^3^H]noradrenaline (specific activity, 42.6 Ci/mmol) in culture medium containing 1 mM ascorbic acid. Thereafter, these discs were introduced into small chambers and superfused with a solution consisting of (in millimolars) NaCl, 120; KCl, 6.0; CaCl_2_, 2.0; MgCl_2_, 2.0; glucose, 20; HEPES, 10; fumaric acid, 0.5; Na-pyruvate, 5.0; and ascorbic acid, 0.57; adjusted to pH 7.4 with NaOH. Superfusion was performed at 25 °C at a rate of about 1.0 ml/min. Collection of 4-min superfusate fractions was started after a 60-min washout period to remove excess radioactivity.

To investigate noradrenaline release evoked by oxotremorine M, the muscarinic agonist was included in the superfusion buffer for 2 min, unless indicated otherwise. For comparison, tritium overflow was also elicited by the application of 60 monophasic rectangular electrical pulses (0.5 ms, 60 mA, 50 V/cm) delivered at a frequency of 1.0 Hz. Modulatory agents, i.e. PKC inhibitors, ion channel blockers, furosemide or bumetanide were present from minute 50 of superfusion (i.e. 10 min prior to the start of sample collection) onward. The radioactivity remaining in the cells after finishing the experiments was extracted by immersion of the discs in 2 % (*v*/*v*) perchloric acid. Radioactivity in extracts and collected fractions was determined by liquid scintillation counting (Perkin Elmer Tri-Carb 2800 TR). Radioactivity released in response to electrical field stimulation from rat sympathetic neurons after labelling with tritiated noradrenaline under conditions similar to those of this study had been shown to consist mainly of the authentic transmitter and to contain only small amounts (<15 %) of metabolites [35]. Therefore, the outflow of tritium as determined here was assumed to reflect the release of noradrenaline and not that of metabolites.

The spontaneous (unstimulated) rate of [^3^H] outflow was obtained by expressing the radioactivity of a collected fraction as a percentage of the total radioactivity in the cultures at the beginning of the corresponding collection period. Stimulation-evoked tritium overflow was calculated as the difference between the total [^3^H] outflow during and after stimulation and the estimated basal outflow which was assumed to decline linearly throughout experiments. Therefore, basal outflow during periods of stimulation was assumed to equate to the arithmetic mean of the samples preceding and those following stimulation, respectively. The difference between the total and the estimated basal outflow was expressed as a percentage of the total radioactivity in the cultures at the beginning of the respective stimulation (% of total radioactivity). The amount of electrically or oxotremorine M-evoked tritium release may vary considerably between different SCG preparations [23]. Therefore, tritium overflow in the presence of release-altering agents, such as PKC, transporter or channel inhibitors, was always compared with that obtained within the same SCG preparation in the presence of solvent. To directly compare the effects of different modulatory agents upon electrically and oxotremorine M-evoked overflow, respectively, the values obtained in the presence of these modulators were expressed as the percentage of the corresponding values in the presence of solvent within the same preparation.

### Statistics

All values are the arithmetic means ± standard error of the mean. *n* values reflect single cells in electrophysiological experiments and numbers of cultures in radiotracer release experiments. Statistical significance of differences between two groups was determined with the Mann–Whitney test. Statistical significance of differences between multiple groups was performed with the Kruskal–Wallis tests followed by Dunn’s multiple comparison tests. Values of *p* <0.05 were considered as indicating statistical significance.

### Materials

(−)-[Ring-2,5,6-^3^H]noradrenaline was obtained from PerkinElmer (Vienna, Austria); amphotericin B, gramicidin D, oxotremorine M, staurosoporine, 12-(2-cyanoethyl)-6,7,12,13-tetrahydro-13-methyl-5-oxo-5H-indolo[2,3-a]pyrrolo[3,4-c]carbazole (Gö6976), 3-[1-[3-(dimethylamino)propyl]-5-methoxy-1H-indol-3-yl]-4-(1H-indol-3-yl)-1H-pyrrole-2,5-dione (Gö 6983), bisindolylmaleimide I (GF 109203 X), phorbol-12-myristate 13-acetate, 10,10-bis(4-pyridinylmethyl)-9(10H)-anthracenone (XE 991), disodium-4-acetamido-4′-isothiocyanato-stilbene-2,2′-disulfonic acid (SITS), niflumic acid and pertussis toxin from Sigma; 6-(1,1-dimethylethyl)-2-[(2-furanylcarbonyl)amino]-4,5,6,7-tetrahydrobenzo[*b*]thiophene-3-carboxylic acid (CaCCinh-A01) and 2-[(5-ethyl-1,6-dihydro-4-methyl-6-oxo-2-pyrimidinyl)thio]-*N*-[4-(4-methoxyphenyl)-2-thiazolyl]acetamide (T16Ainh-A01) from Tocris (Bristol, UK); and tetrodotoxin was from Latoxan (Rosans, France). Water-insoluble drugs were first dissolved in DMSO and then diluted into buffer to yield final DMSO concentrations of up to 0.3 %, which were also included in control solutions.

## Results

### Depolarisation of SCG neurons via M_1_ receptors is not matched with the inhibition of K_v_7 channels

**Fig. 1:**
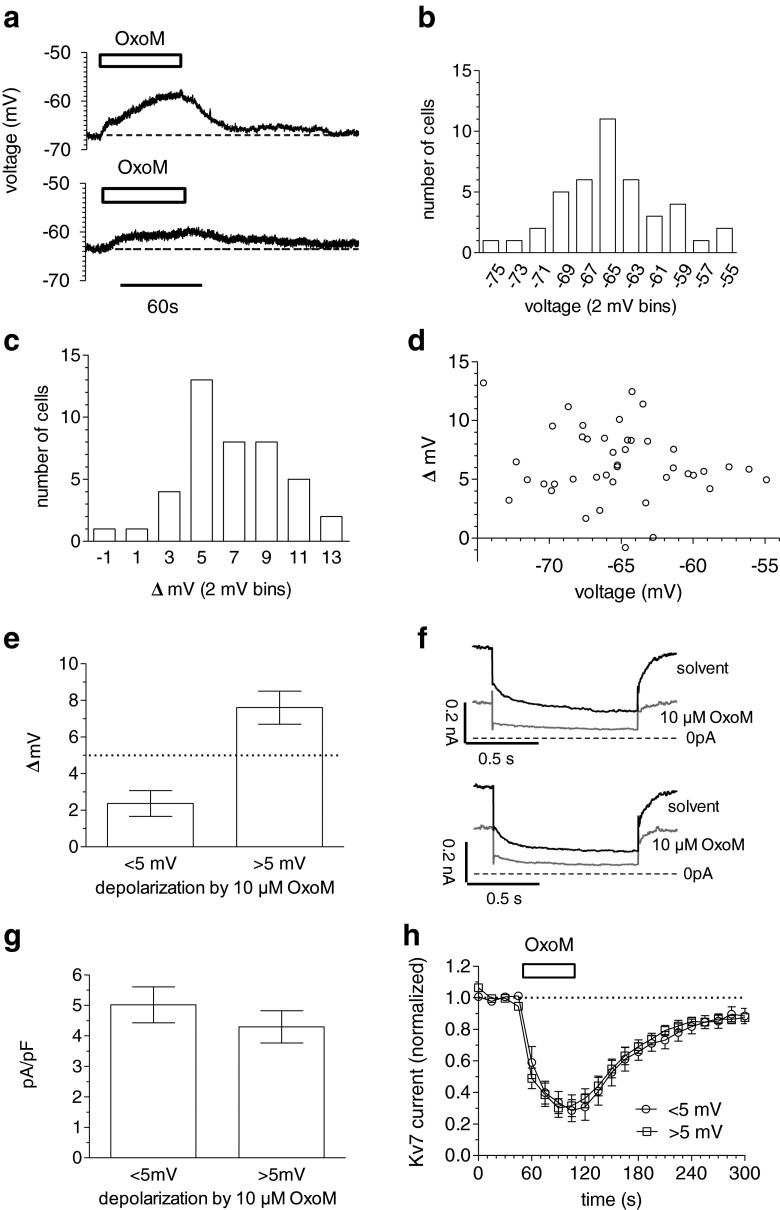
Comparison of depolarisation and K_v_7 channel inhibition by oxotremorine M. Membrane potential and currents through K_v_7 channels in SCG neurons were recorded in current-clamp and voltage-clamp mode, respectively, using the amphotericin B-perforated patch technique. **a** Time course of membrane voltage in two different SCG neurons; oxotremorine M (*OxoM*, 10 μM) was present, as indicated by the *bars*. **b** Frequency distribution of resting membrane potentials (voltage in bins of 2 mV) as determined in 42 SCG neurons. **c** Frequency distribution of changes in membrane potentials (Δ mV in bins of 2 mV) caused by 10 μM oxotremorine M as determined in these 42 SCG neurons. **d** Correlation between resting membrane potential (voltage) and membrane potential changes (Δ mV) caused by 10 μM oxotremorine M in the same 42 SCG neurons; experiments were carried out as shown in (**a**). The Spearman’s coefficient for this correlation is −0.053 (95% confidence interval, −0.3594 to 0.2638). **e** Subset of 16 neurons categorized according to the extent of depolarisation caused by 10 μM oxotremorine M (<5 mV, *n* = 8; >5 mV, *n* = 8); the means of the depolarisation observed in these two groups are shown. **f** Subsequently, currents through K_v_7 channels were recorded by holding these 16 cells at a voltage of −30 mV and by applying hyperpolarisations to −55 mV once every 15 s. The *traces* show current responses of two neurons, one out of each of these two categories. **g** Mean values of densities of deactivation currents caused by the steps from −30 to −55 mV in the neurons from both categories (*n* = 8). **h** Time course of deactivation current amplitudes caused by the steps from −30 to −55 mV in the neurons from both categories; oxotremorine M (*OxoM*, 10 μM) was present, as indicated by the *bar* (*n* = 8)

In order to evaluate the relation between the depolarisation of SCG neurons and the inhibition of K_v_7 channels, both through the activation of M_1_ receptors by oxotremorine M, an initial set of 42 neurons was investigated. The values of resting membrane potentials in these neurons ranged between −55 and −75 mV. Changes in membrane potential caused by 10 μM oxotremorine M varied between −1 and +13 mV. There was no correlation between these values of resting membrane potential and the changes induced by the muscarinic agonist (Fig. 1a–d). In a subset of neurons with oxotremorine M-induced depolarisations of either less (*n* = 8) or more (*n* = 8) than 5 mV, currents through K_v_7 channels were determined subsequently to the current-clamp measurements (Fig. 1e, f). The densities of K_v_7 deactivation currents (triggered by hyperpolarisations from −30 to −55 mV) were comparable in these two groups of neurons (Fig. 1g). Likewise, the extent as well as the time course of current inhibition by 10 μM oxotremorine M for these two sets of neurons were indiscernible (Fig. 1h). We therefore concluded that mechanisms other than the inhibition of K_v_7 channels also contribute to the depolarisation caused by oxotremorine M. As the extent of oxotremorine M-induced depolarisation varies considerably between single neurons (Fig. 1b), the underlying signalling cascade was investigated only in neurons that displayed depolarisations of at least 5 mV.

### Activation of PKC contributes to the depolarisation of SCG neurons by oxotremorine M, but not to the inhibition of K_v_7 channels

**Fig. 2:**
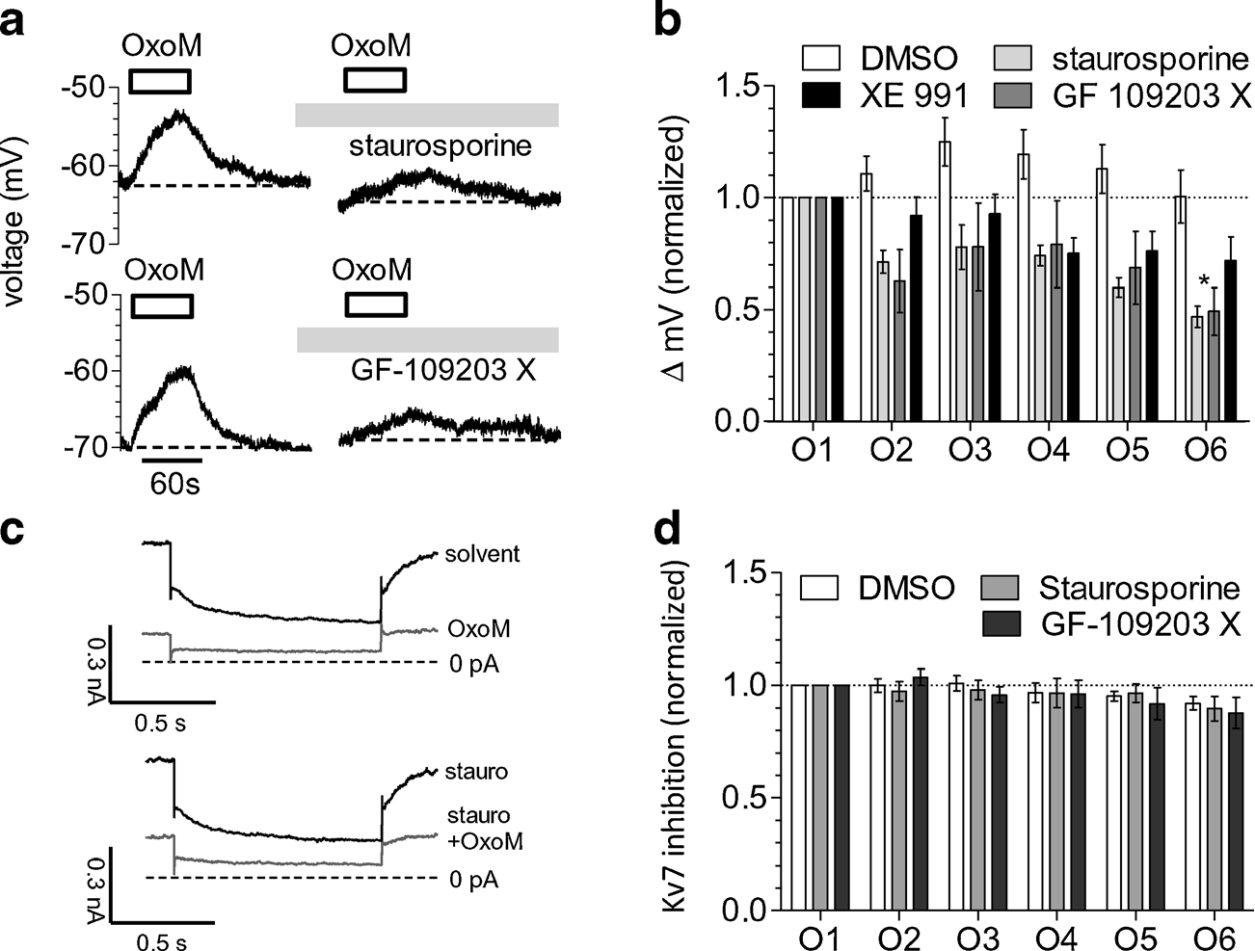
Effects of PKC inhibitors on depolarisation and K_v_7 channel inhibition by oxotremorine M. Membrane potential and currents through K_v_7 channels in SCG neurons were recorded in current-clamp and voltage-clamp mode, respectively, using the amphotericin B-perforated patch technique. Oxotremorine M (*OxoM*, 10 μM) was present for six periods of 60 s each; these periods of oxotremorine M application were separated by 3-min intervals. From minute 2 after the first oxotremorine M application onward, PKC inhibitors, XE 991, or solvent (0.1 % DMSO) was present throughout the remaining measurements. **a** Time course of membrane voltage in two different SCG neurons during the first and the sixth exposure to oxotremorine M (*OxoM*, 10 μM); the agonist was present, as indicated by the *bars*. After the first oxotremorine M exposure, either 1 μM staurosporine or 1 μM GF 109203 X was present. **b** Changes in membrane voltage (Δ mV) caused by these six oxotremorine M applications (*O1*–*O6*) in the presence of DMSO, staurosporine, GF 109203 X or XE 991 (3 μM); the values of these six depolarisations were normalized to the value of the first one (*n* = 6). *Significant difference between the four values at O6 (*p* < 0.05, Kruskal–Wallis test). **c** Current responses of one neuron that was clamped at a voltage of −30 mV and hyperpolarised to −55 mV once every 15 s and that has been exposed to 1 μM staurosporine. The traces were obtained before (solvent) and during (*OxoM*) the first application (*O1*) of 10 μM oxotremorine M as well as before (*stauro*) and during (*stauro+OxoM*) the sixth application (*O6*) of oxotremorine M. **d** Changes in K_v_7 inhibition (quantified by deactivation current amplitudes) caused by these six oxotremorine M applications (*O1*–*O6*) in the presence of either DMSO, staurosporine or GF 109203 X; these six values of K_v_7 inhibition were normalized to the value of the first one (*n* = 6–9)

The activation of PKC contributes to the depolarisation of SCG neurons through B_2_ bradykinin receptors [34]. Therefore, various kinase inhibitors were tested for their effect on depolarisations triggered by 10 μM oxotremorine M (which was applied repeatedly once every 4 min; Fig. 2a, b). Staurosporine (1 μM), a broad-spectrum kinase inhibitor, increasingly reduced oxotremorine M-evoked depolarisations over a time period of 20 min (Fig. 2a, b): initial depolarisations amounted to 8.4 ± 1.1 mV; after 20 min of staurosporine exposure, this value had decreased to 4.0 ± 0.75 mV (*n* = 6, *p* < 0.01, Kruskal–Wallis test). An analogous effect was observed when the PKC inhibitor GF 109203 X (1 μM) was used instead (Fig. 2a, b). After 20 min of its presence, the extent of depolarisation caused by oxotremorine M had decreased from 6.4 ± 0.7 to 3.0 ± 0.6 mV (*n* = 6, *p* < 0.01, Kruskal–Wallis test). However, the solvent (0.1 % DMSO) did not cause significant changes when present as long as the kinase inhibitors, and the depolarisations amounted to 7.0 ± 0.9 mV in the beginning and to 6.9 ± 1.1 mV (*n* = 6, *p* > 0.1, Kruskal–Wallis test) 20 min later. For a comparison with PKC inhibitors, the effects of the K_v_7 channel blocker XE 991 (3 μM) were investigated in an analogous manner: in its presence, the oxotremorine M-induced depolarisations decreased from 8.5 ± 1.0 to 3.8 ± 0.9 mV (*n* = 6, *p* < 0.05, Kruskal–Wallis test). When directly comparing the effects of staurosporine, GF 109203 X, XE 991 and DMSO by normalizing the oxotremorine M-induced depolarisations to the respective first value, the values after 20 min exposure to staurosporine, GF 109203 X or XE 991 were smaller than those after exposure to the solvent (Fig. 2b). Thus, the PKC inhibitors staurosporine and GF 109203 X significantly attenuated the depolarising action of the muscarinic agonist, as did the K_v_7 channel blocker XE 991.

Activation of PKC may also contribute to the inhibition of K_v_7 channels via M_1_ muscarinic receptors [15]. Hence, the parallel effects described above might occur through actions converging at the level of K_v_7 channels. To clarify whether the PKC inhibitors interfered with the muscarinic inhibition of K_v_7 channels, deactivation currents through K_v_7 channels were determined, and 10 μM oxotremorine M was applied once every 4 min again. In the presence of the solvent (DMSO; Fig. 2c), the inhibition of deactivation amplitudes by the agonist declined from 77.9 ± 5.7 to 71.1 ± 4.9 % (*n* = 9). Likewise, in the presence of 1 μM staurosporine and 1 μM GF 109203 X, this inhibition decreased from 81.9 ± 5.2 to 72.2 ± 3.0 % (*n* = 6) and from 68.1 ± 11.1 to 56.1 ± 8.1 % (*n* = 7), respectively (Fig. 2c, d). None of these changes in K_v_7 inhibition were statistically significant (*p* > 0.05, Kruskal–Wallis test). A direct comparison of normalized inhibition values did not reveal any differences between staurosporine, GF 109203 X or solvent (Fig. 2d). Hence, in these experiments with repeated K_v_7 inhibition by 10 μM oxotremorine M, the employed PKC inhibitors did not cause any alteration.

### Inhibition or downregulation of PKC attenuates oxotermorine M-induced noradrenaline release

**Fig. 3:**
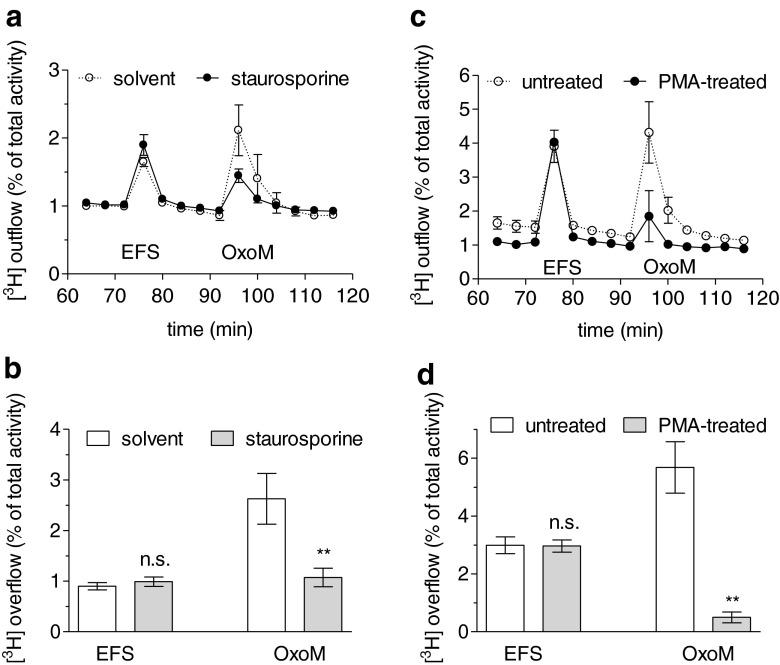
Effect of PKC inhibition on noradrenaline release evoked by electrical field stimulation or oxotremorine M. Cultures of SCG were labelled with [^3^H]noradrenaline and superfused, and subsequent to a 60-min washout period, 4-min fractions of superfusate were collected. Sixty monophasic rectangular pulses (0.5 ms, 60 mA, 50 V/cm) were applied in minute 73, and oxotremorine M (10 μM) was present in minutes 93 and 94. From minute 50 of superfusion onward, the buffer contained either solvent (0.1 % DMSO) or 1 μM staurosporine. Alternatively, cultures had been treated with either 0.1 % DMSO (untreated) or 1 μM phorbol-12-myristate-13-acetate (PMA-treated) for 24 h. **a** Time course of [^3^H] outflow as a percentage of radioactivity in the cells in the presence of either solvent (*clear circles*) or staurosporine (*black circles*, *n* = 3). **b** Summary of the effect of 1 μM staurosporine on [^3^H] overflow evoked by electrical field stimulation (*EFS*) or oxotremorine M (*OxoM*, *n* = 8–9). **c** Time course of [^3^H] outflow as a percentage of radioactivity in the cells which had either been treated with phorbol-12-myristate-13-acetate (*black circles*) or had remained untreated (*clear circles*, *n* = 3). **d** Summary of the effect of phorbol-12-myristate-13-acetate treatment on [^3^H] overflow evoked by electrical field stimulation (*EFS*) or oxotremorine M (*OxoM*, *n* = 12). **p < 0.01 (vs. solvent and untreated). *n.s.* no significance

To investigate the signalling cascade of oxotremorine M-induced depolarisations not only in single cells, which are quite heterogeneous in this response, a large population of neurons was investigated simultaneously. This was achieved by loading the neurons with [^3^H]noradrenaline and by subsequently stimulating the overflow of radioactivity by this muscarinic agonist. In such experiments, oxotremorine M, at concentrations <100 μM, triggers overflow of radioactivity through the selective activation of M_1_ receptors [23]. To control for effects of PKC inhibitors unrelated to the signalling cascade of M_1_ receptors, tritium overflow was also elicited by electrical field stimulation. Staurosporine (1 μM) did not alter tritium overflow triggered by electrical fields, but reduced that evoked by oxotremorine M by more than 50 % (Fig. 3a, b). Exposure of SCG cultures to phorbol-12-myristate-13-acetate for 24 h downregulates all but atypical PKC isoforms [34]. In cultures treated in that way, electrically evoked release was the same as in untreated sister cultures. However, oxotremorine M-induced tritium overflow in phorbol-12-myristate-13-acetate-treated cultures amounted to only 10 % of that in untreated cultures (Fig. 3c, d).

**Fig. 4:**
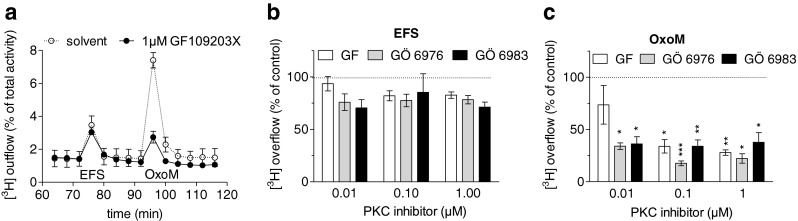
Effects of subtype preferring PKC inhibitors on noradrenaline release evoked by electrical field stimulation or oxotremorine M. Cultures of SCG were labelled with [^3^H]noradrenaline and superfused, and subsequent to a 60-min washout period, 4-min fractions of superfusate were collected. Sixty monophasic rectangular pulses (0.5 ms, 60 mA, 50 V/cm) were applied in minute 73, and oxotremorine M (10 μM) was present in minutes 93 and 94. From minute 50 of superfusion onward, the buffer contained either solvent (0.1 % DMSO) or 0.01 to 1 μM of GF 109203 X (*GF*), Gö 6976 or Gö 6983. **a** Time course of [^3^H] outflow as a percentage of radioactivity in the cells in the presence of either solvent (*clear circles*) or 1 μM of GF 109203 X (*black circles*, *n* = 3). **b** Summary of the effects of the indicated concentrations of PKC inhibitors on [^3^H] overflow evoked by electrical field stimulation (*EFS*). Overflow in the presence of the inhibitors is depicted as a percentage of the overflow in the presence of solvent (% of control). **c** Summary of the effects of the indicated concentrations of PKC inhibitors on [^3^H] overflow evoked by oxotremorine M (*OxoM*). Overflow in the presence of the inhibitors is depicted as a percentage of the overflow in the presence of solvent (% of control). In (**b**) and (**c**), *n* = 6–9. *p < 0.05; **p < 0.01; ***p < 0.001 (vs. solvent)

Together, the above results indicate that some PKC isoforms, with the exception of atypical ones, are involved in the excitation of SCG neurons via M_1_ receptors. To further elaborate which PKC subtypes may contribute, GF 109203 X and related PKC inhibitors (GÖ 6976 and GÖ 6983) with divergent subtype preferences [37] were employed. None of these drugs caused significant alterations in electrically induced tritium overflow (Fig. 4b). In contrast, at 0.01 μM, GÖ 6976 and GÖ 6983, but not GF 109203 X, significantly diminished oxotremorine M-evoked overflow, and at higher concentrations, all the PKC inhibitors shared this effect (Fig. 4c). Thus, with respect to the inhibition of noradrenaline release caused by oxotremorine M, GÖ 6976 and GÖ 6983 were more potent than GF 109203 X.

### Cl^−^ conductances contribute to the depolarisation of SCG neurons by oxotremorine M

**Fig. 5:**
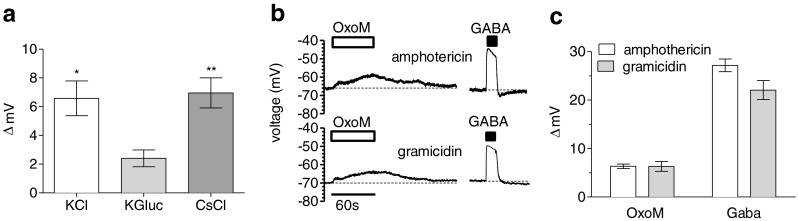
Effects of Cl^−^ and ionophore substitution on depolarisations by oxotremorine M. Membrane potential in SCG neurons was recorded in current-clamp mode using the amphotericin B- or gramicidin D-perforated patch technique. **a** Extent of depolarisations elicited by 10 μM oxotremorine M in amphotericin B-perforated patch recordings with pipette solutions containing 75 mM K_2_SO_4_ plus 55 mM KCl, 55 mM potassium gluconate (*KGluc*) or 55 mM CsCl (*n* = 5–7). *p < 0.05; **p < 0.01 (vs. potassium gluconate, Kruskal–Wallis test). **b** Original current-clamp traces using either amphotericin B (*upper traces*) or gramicidin D (*lower traces*). 10 μM oxotremorine M (*OxoM*) or 10 μM GABA was present, as indicated by the *bars*. **c** Extent of depolarisations elicited by 10 μM oxotremorine M or 10 μM GABA in either amphotericin B- or gramicidin D-perforated patch recordings (*n* = 5–7)

To elucidate the ionic basis of oxotremorine M-induced depolarisations, the composition of the pipette solution was changed by replacing 55 mM KCl by equimolar concentrations of either CsCl or potassium gluconate. The resting membrane potentials determined with these three different pipette solutions were −65.2 + 2.4 mV (KCl, *n* = 7), −77.7 + 3.3 mV (K-gluconate, *n* = 5) and −62.8 + 1.9 mV (CsCl, *n* = 5). Whilst changes in membrane voltage caused by 10 μM oxotremorine M were not affected by alterations in K^+^, the reduction of intracellular Cl^-^ clearly reduced the depolarising response (Fig. 5a).

Since this result suggested that Cl^−^ was the relevant ion, the effects of oxotremorine M were compared with those of GABA. As in the case of oxotremorine M, 10 μM GABA depolarised the neurons. The depolarisations caused by GABA developed instantaneously and then decayed during the presence of the transmitter, whereas the oxotremorine M-induced depolarisations developed slowly during the 1-min exposure towards the agonist (Fig. 5b). In perforated patch recordings with amphotericin B, intracellular anion concentrations depend on those of the pipette solution (see above). This disruption of the intracellular anion homeostasis can be prevented by using gramicidin D instead of amphotericin B [2]. With our standard pipette solution containing 55 mM KCl, depolarisations caused by 10 μM oxotremorine M were the same, whether amphotericin B or gramicidin D was used as the ionophore, and the same was true for GABA-evoked changes in membrane voltage (Fig. 5b, c). Thus, depolarisations caused by M_1_ receptor activation appear to rely on high intracellular Cl^−^ concentrations and, hence, on a Cl^−^ conductance.

### Alterations in extracellular Cl^−^ affect noradrenaline release induced by either oxotremorine M or GABA

**Fig. 6:**
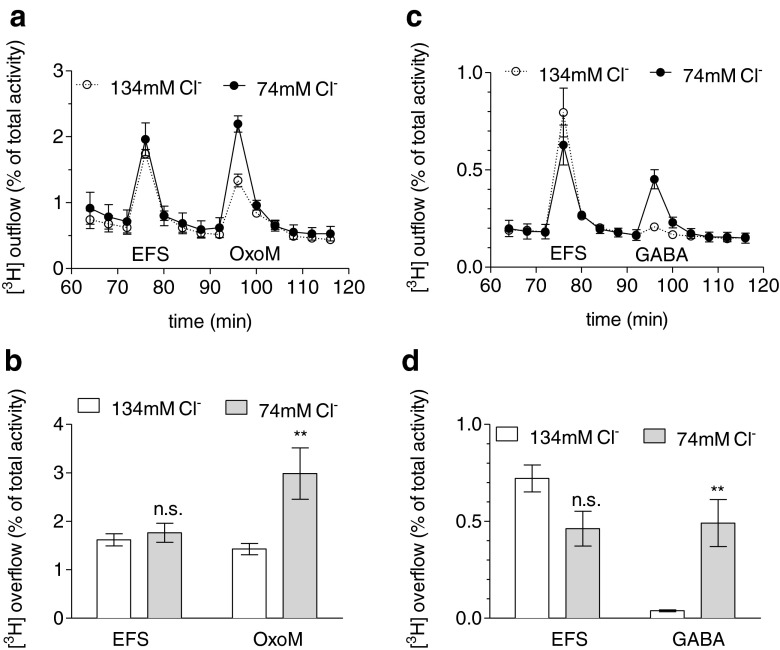
Effects of Cl^−^ substitution on noradrenaline release evoked by electrical field stimulation, oxotremorine M or GABA. Cultures of SCG were labelled with [^3^H]noradrenaline and superfused, and subsequent to a 60-min washout period, 4-min fractions of superfusate were collected. Sixty monophasic rectangular pulses (0.5 ms, 60 mA, 50 V/cm) were applied in minute 73, and oxotremorine M (10 μM) (**a**, **b**) or GABA (10 μM) (**c**, **d**) was present in minutes 93 and 94. From minute 50 of superfusion onward, the buffer contained 134 or 74 mM Cl^−^ (the lacking Cl^−^ was replaced by gluconate). **a**, **c** Time course of [^3^H] outflow as a percentage of radioactivity in the cells (*n* = 3). **b** Summary of the amount of [^3^H] overflow evoked by electrical field stimulation (*EFS*) and oxotremorine M (*OxoM*), respectively (*n* = 12). **d** Summary of the amount of [^3^H] overflow evoked by electrical field stimulation (*EFS*) and GABA, respectively (*n* = 10–12). **p < 0.01 (vs. 134 mM Cl^−^). *n.s.* no significance

To explore the role of Cl^−^ conductances in the stimulatory action of oxotremorine M on noradrenaline release, [^3^H] overflow was triggered by this agonist either in quasi-physiological solution (containing 134 mM Cl^−^) or in a solution in which 60 mM NaCl had been replaced by 60 mM sodium gluconate. In these two different solutions, tritium overflow triggered by electrical field stimulation was essentially the same (and if anything, reduced in the presence of sodium gluconate rather than enhanced; Fig. 6d). Oxotremorine M-evoked overflow, however, was significantly enhanced when extracellular Cl^−^ had been reduced (Fig. 6a, b). For comparison, cultures were exposed to 10 μM GABA instead of the same concentration of the muscarinic agonist. As expected, GABA-induced overflow was also enhanced by lowering extracellular Cl^−^ (Fig. 6c, d). Thus, the stimulation of noradrenaline release from SCG neurons through the activation of M_1_ receptors depends on the extracellular Cl^−^ concentration.

### Cl^−^ channel blockers diminish the depolarisation of SCG neurons by oxotremorine M

**Fig. 7:**
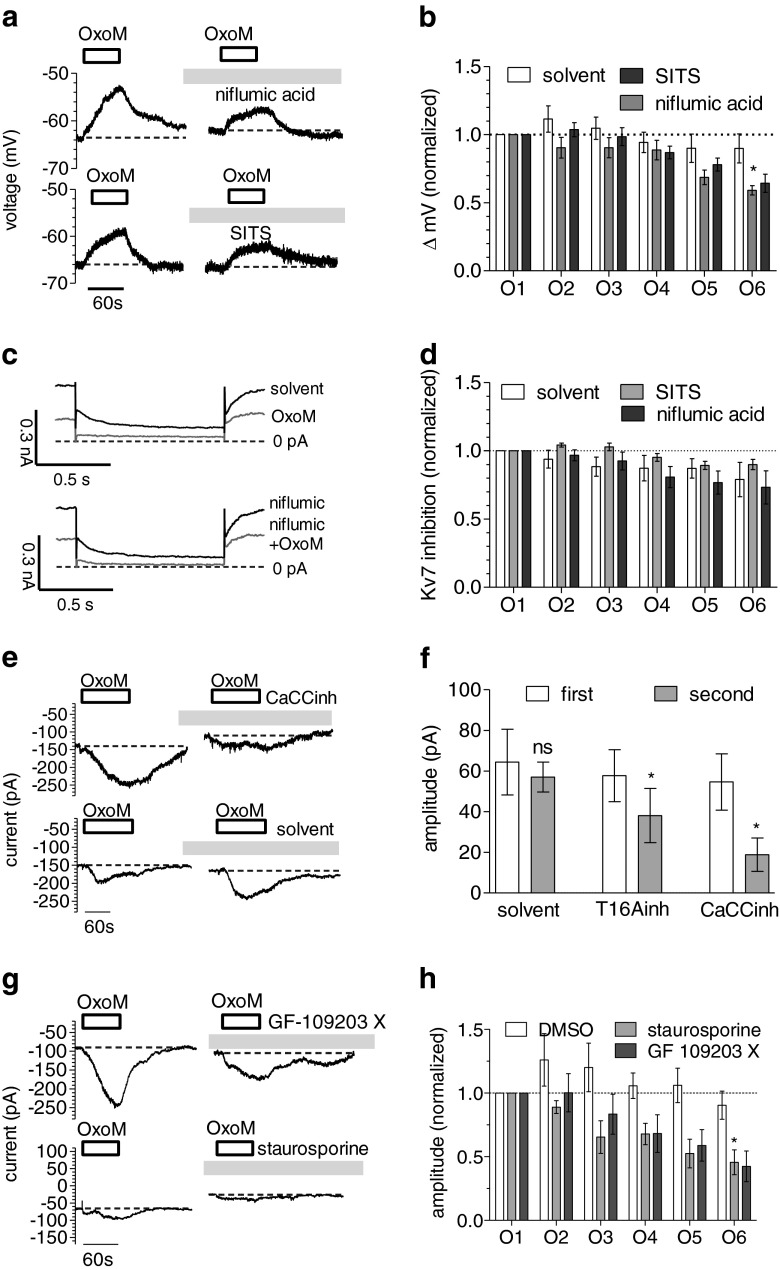
Effects of Cl^−^ channel blockers on depolarisations, K_v_7 channel inhibition and inward currents induced by oxotremorine M. Membrane potential and currents in SCG neurons were recorded in current-clamp and voltage-clamp mode, respectively, using the amphotericin B-perforated patch technique. **a**–**d**, **g**–**h** Oxotremorine M (OxoM, 10 μM) was present for six periods of 60 s each; these periods of oxotremorine M application were separated by 3-min intervals. From minute 2 after the first oxotremorine M application onward, Cl^−^ channel blockers or solvent was present throughout the remaining measurement. **a** Time course of membrane voltage in two different SCG neurons during the first and the sixth exposure to oxotremorine M (*OxoM*, 10 μM); the agonist was present, as indicated by the *bars*. After the first oxotremorine M exposure, either 300 μM niflumic acid or 300 μM SITS was present. **b** Changes in membrane voltage (Δ mV) caused by these six oxotremorine M applications (*O1*–*O6*) in the presence of either solvent, niflumic acid or SITS; the values of these six depolarisations were normalized to the value of the first one (*n* = 7). *Significant difference between the three values at O6 (*p* < 0.05, one-way Kruskal–Wallis test). **c** Current responses of one neuron that was clamped at a voltage of −30 mV and hyperpolarised to −55 mV once every 15 s and that has been exposed to 300 μM niflumic acid. The traces were obtained before (*solvent*) and during (*OxoM*) the first application (*O1*) of 10 μM oxotremorine M as well as before (*niflumic*) and during (*niflumic+OxoM*) the sixth application (*O6*) of oxotremorine M. **d** Changes in K_v_7 inhibition (quantified by deactivation current amplitudes) caused by these six oxotremorine M applications (*O1*–*O6*) in the presence of either solvent, niflumic acid or SITS; these six values of K_v_7 inhibition were normalized to the value of the first one (*n* = 7). **e**, **f** Currents recorded at a holding potential of −65 mV. Oxotremorine M (10 μM) was present for two periods of 120 s each; these periods of oxotremorine M application were separated by 5-min intervals. From minute 2 after the first oxotremorine M application onward, Cl^−^ channel blockers or solvent was present throughout the remaining measurement. **e** Time course of membrane currents in two different SCG neurons during the first and the second exposure to oxotremorine M (*OxoM*, 10 μM); the agonist was present, as indicated by the *bars*. After the first oxotremorine M exposure, either 3 μM CaCCinh or 0.1 % DMSO (solvent) was present. **f** Current amplitudes caused by the first and the second oxotremorine M application in the presence of either solvent, 3 μM CaCCinh or 3 μM T16Ainh (*n* = 6). *p < 0.05 (Wilcoxon matched-pairs signed-rank test). **g** Time course of membrane currents in two different SCG neurons during the first and the sixth exposure to oxotremorine M (*OxoM*, 10 μM); the agonist was present, as indicated by the *bars*. After the first oxotremorine M exposure, either 1 μM GF 109203 X or 1 μM staurosporine was present. **h** Changes in the amplitudes of the currents caused by these six oxotremorine M applications (*O1*–*O6*) in the presence of either solvent, 1 μM GF 109203 X or 1 μM staurosporine; the values of these six current amplitudes were normalized to the value of the first one (*n* = 7). *Significant difference between the three values at O6 (*p* < 0.05, Kruskal–Wallis test)

The above results hint to a role of Cl^−^ conductances in the excitatory action of oxotremorine M. There is a large number of different voltage- and Ca^2+^-gated Cl^−^ channels, but only a comparably low number of relatively unselective blockers [12, 32]. Two frequently used representatives of these blockers are SITS and niflumic acid, which were tested for their effects on depolarisations triggered by 10 μM oxotremorine M (which was applied repeatedly as in Fig. 2). As the effects of Cl^−^ channel blockers on the channels are complex (with voltage-dependent enhancing and decreasing activities) and develop slowly [33], these agents were applied for prolonged periods of time. In the presence of 300 μM niflumic acid or SITS (Fig. 7a), oxotremorine M-induced depolarisations decreased from 7.4 + 0.8 to 4.4 + 0.6 mV (*n* = 7, *p* < 0.05, Kruskal–Wallis test). An equivalent decline was observed with 300 μM SITS (Fig. 7a): the extent of depolarisation caused by oxotremorine M fell from 6.6 + 0.4 to 4.2 + 0.5 mV (*n* = 7, *p* < 0.001, Kruskal–Wallis test). However, the solvent did not cause significant changes, and the depolarisations amounted to 8.2 + 0.8 mV in the beginning and to 7.2 + 0.9 mV (*n* = 7, *p* > 0.1, Kruskal–Wallis test) at the end of experiments. When directly comparing these changes by normalizing the oxotremorine M-induced depolarisations, the values after exposure to either SITS or niflumic acid were significantly smaller than those obtained in the solvent (Fig. 7b). Thus, the two Cl^−^ channels blockers significantly attenuated the depolarising action of the muscarinic agonist.

To reveal whether these channel blockers might also affect K_v_7 channels or their inhibition via muscarinic receptors, currents through these latter channels were determined again. In the presence of 300 μM niflumic acid (Fig. 2c) and 300 μM SITS, the inhibition of K_v_7 deactivation currents decreased from 89.7 + 4.5 to 68.2 + 12.4 % (*n* = 7) and from 87.0 + 2.5 to 78.1 + 3.5 % (*n* = 7), respectively. In the solvent, a similar trend was observed and the oxotremorine M-induced inhibition was 90.8 + 4.2 % in the beginning and 74.8 + 12.5 % (*n* = 7) at the end of recordings. All these changes in K_v_7 inhibition were statistically non-significant. Furthermore, a direct comparison of normalized inhibition values did not reveal any differences between niflumic acid, SITS and solvent (Fig. 7d).

### Inward currents induced by oxotremorine M are attenuated by blockers of Ca^2+^-activated Cl^−^ channels and by PKC inhibitors

To find out whether oxotremorine M can induce depolarising currents, neurons were clamped to −65 mV and the M_1_ receptor agonist was applied for periods of 2 min. The potential of −65 mV was chosen as this was the median value of membrane potentials as determined in current-clamp experiments (Fig. 1b). Moreover, at this voltage, K_v_7 channels of SCG neurons are not activated [23]. In the presence of 10 μM oxotremorine M, inward currents developed slowly and reached a maximum after 30 s to 2 min (Fig. 7e). Maximal current amplitudes ranged between 20 and 120 pA.

As these currents were triggered by the activation of M_1_ receptors, but not by changes in membrane voltage, it appeared straightforward to assume that they were carried by Ca^2+^-activated rather than voltage-gated Cl^−^ channels. Hence, currents were induced again by a second application of oxotremorine M to the very same cells in the presence of CaCCinh-A01 or T16Ainh-A01, two selective blockers of different Ca^2+^-activated Cl^−^ channels [31]. The muscarinic agonist triggered currents of similar amplitudes again when reapplied in the presence of the solvent (0.1 % DMSO). In contrast, in the presence of 3 μM CaCCinh-A01 or 3 μM T16Ainh-A01, current amplitudes caused by the second oxotremorine M application were reduced significantly (Wilcoxon matched-pair signed-rank test; Fig. 7e, f).

To reveal whether the triggering of these currents by oxotremorine M does also involve PKC, the agonist was applied repeatedly in the presence of staurosporine, GF 109203 X or solvent (as shown for depolarisations in Fig. 2a, b). Staurosporine (1 μM) increasingly reduced oxotremorine M-evoked currents: initial amplitudes amounted to 64.8 + 20.1 pA, and these were reduced to 36.5 + 19.6 pA (*n* = 7, *p* < 0.05, Kruskal–Wallis test) 20 min later. Likewise, when the PKC inhibitor GF 109203 X (1 μM) was used, amplitudes decreased from 77.8 + 14.4 to 33.8 + 10.6 pA (*n* = 7, *p* < 0.05, Kruskal–Wallis test). However, the solvent (0.1 % DMSO) did not cause significant changes, and the amplitudes amounted to 95.6 + 37.8 and to 71.3 + 26.4 pA (*n* = 7, *p* > 0.1, Kruskal–Wallis test) in the beginning and at the end, respectively (Fig. 7g, h).

### Blockers of Cl^−^ channels and Cl^−^ transporters reduce noradrenaline release induced by oxotremorine M

**Fig. 8:**
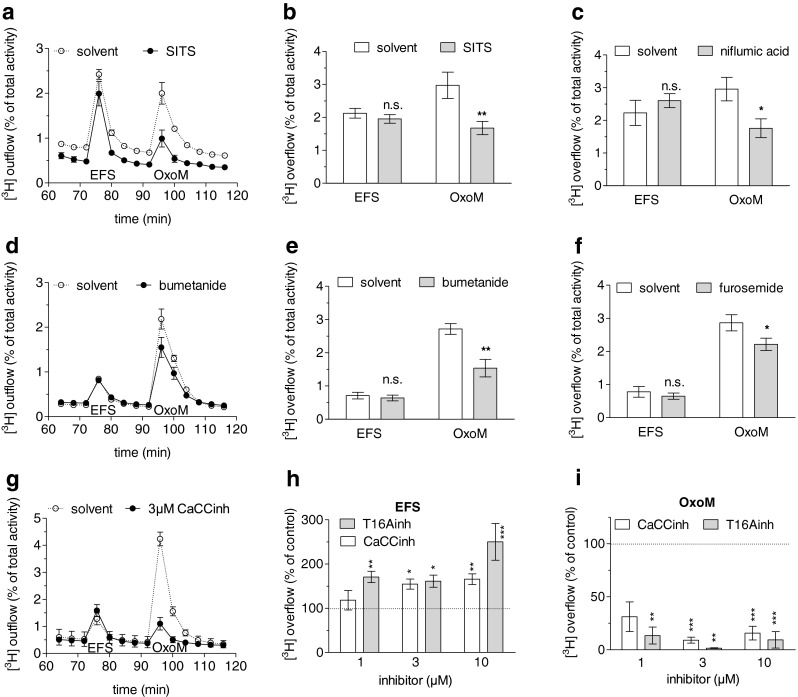
Effects of Cl^−^ channel blockers and inhibitors of Cl^−^ transporters on noradrenaline release evoked by electrical field stimulation or oxotremorine M. Cultures of SCG were labelled with [^3^H]noradrenaline and superfused, and subsequent to a 60-min washout period, 4-min fractions of superfusate were collected. Sixty monophasic rectangular pulses (0.5 ms, 60 mA, 50 V/cm) were applied in minute 73, and oxotremorine M (10 μM) was present in minutes 93 and 94. From minute 50 of superfusion onward, the buffer contained solvent, 300 μM SITS, 300 μM niflumic acid, 300 μM bumetanide, 300 μM furosemide, CaCCinh or T16Ainh, the latter two at concentrations of 1, 3 or 10 μM. **a** Time course of [^3^H] outflow as a percentage of radioactivity in the cells in the presence of either solvent (*clear circles*) or SITS (*black circles*, *n* = 3). **b** Summary of the effect of 300 μM SITS on [^3^H] overflow evoked by electrical field stimulation (*EFS*) or oxotremorine M (*OxoM*, *n* = 18). **c** Summary of the effect of 300 μM niflumic acid on [^3^H] overflow evoked by electrical field stimulation (*EFS*) or oxotremorine M (*OxoM*, *n* = 9). **d** Time course of [^3^H] outflow as a percentage of radioactivity in the cells in the presence of either solvent (*clear circles*) or bumetanide (*black circles*, *n* = 3). **e** Summary of the effect of 300 μM bumetanide on [^3^H] overflow evoked by electrical field stimulation (*EFS*) or oxotremorine M (*OxoM*, *n* = 12). **f** Summary of the effect of 300 μM furosemide on [^3^H] overflow evoked by electrical field stimulation (*EFS*) or oxotremorine M (*OxoM*, *n* = 12). *p < 0.05; **p < 0.01 (vs. the respective value obtained in solvent). **g** Time course of [^3^H] outflow as a percentage of radioactivity in the cells in the presence of either solvent (*clear circles*) or 3 μM CaCCinh (*black circles*, *n* = 3). **h** Summary of the effects of the indicated concentrations of CaCCinh or T16Ainh on [^3^H] overflow evoked by electrical field stimulation (*EFS*). Overflow in the presence of the inhibitors is depicted as a percentage of the overflow in the presence of solvent (% of control). **i** Summary of the effects of the indicated concentrations of CaCCinh or T16Ainh on [^3^H] overflow evoked by oxotremorine M (*OxoM*). Overflow in the presence of the inhibitors is depicted as a percentage of the overflow in the presence of solvent (% of control). In (**h**) and (**i**), *n* = 6–9. *p < 0.05; **p < 0.01; ***p < 0.001 (vs. solvent, Kruskal–Wallis test)

To corroborate the data shown above by an independent approach, SITS and niflumic acid were the first blockers to be tested for their effects on noradrenaline release. These two agents did not affect electrically evoked tritium overflow, but significantly reduced overflow induced by oxotremorine M (Fig. 8a–c).

The release-enhancing effect of reductions in extracellular Cl^−^ concentrations (Fig. 6) points to a role of high intracellular Cl^−^ in the depolarising action of oxotremorine M. Neurons in the peripheral nervous system express Na^+^/K^+^/Cl^−^ co-transporters which mediate Cl^−^ uptake and intracellular Cl^−^ accumulation. These transporters can be blocked by diuretics such as furosemide and bumetanide [19]. In the presence of 300 μM of either of these two drugs, overflow of radioactivity triggered by electrical fields remained unaltered, whereas oxotremorine M-induced overflow was significantly reduced (Fig. 8d–f). Thus, hindrance of Cl^−^ uptake into the neurons selectively diminished the secretagogue action of oxotremorine M.

The results obtained with CaCCinh-A01 or T16Ainh-A01 in electrophysiological experiments were also confirmed with respect to [^3^H]noradrenaline release: both blockers at 1–10 μM did reduce tritium overflow triggered by the muscarinic agonist (Fig. 8g, i). Electrically evoked overflow, however, was not reduced, but rather enhanced by CaCCinh-A01 as well as T16Ainh-A01 in a concentration-dependent manner (Fig. 8g, h).

## Discussion

Transmission in autonomic ganglia involves acetylcholine as the prime transmitter which triggers fast and slow EPSPs mediated by nicotinic and muscarinic receptors, respectively. The slow component of ganglionic transmission has been known to be mediated by an inhibition of K_v_7 channels via M_1_ receptors for more than three decades [9]. In primary cultures of rat SCG neurons, the activation of M_1_ receptors causes depolarisation and ensuing noradrenaline release, and evidence has been presented that suggests these effects to rely on an inhibition of K_v_7 channels as well [23]. However, the present results reveal an additional and novel mechanism of M_1_ receptor-dependent excitation of sympathetic neurons that is independent of K_v_7 channels.

Depolarisations caused by the mAChR agonist oxotremorine M were remarkably variable between single neurons and not related to the levels of resting membrane potential. However, there was no correlation between the extent of depolarisation and either K_v_7 channel current densities or the degree of K_v_7 channel inhibition by oxotremorine M. Furthermore, the K_v_7 channel blocker XE 991 reduced depolarisations caused by this muscarinic agonist, but did not abolish them. Hence, it appeared obvious to search for additional mechanisms involved in the depolarisation of SCG neurons via M_1_ mAChRs.

The activation of M_1_ receptors in sympathetic neurons turns on the entire Gq- and phospholipase C-linked signalling cascade which includes a boost of PKC [26]. Activated PKC may contribute to the muscarinic inhibition of K_v_7 channels [15], but this effect is generally believed to be mainly mediated by the depletion of membrane phosphatidylinositol-4,5-bisphosphate [14]. In accordance with this latter concept, the present experiments did not reveal any effect of PKC inhibitors on the oxotremorine M-induced inhibition of currents though K_v_7 channels. In contrast, staurosporine and GF 109203 X both reduced depolarisations caused by the muscarinic agonist. Moreover, noradrenaline release evoked by oxotremorine M (but not that induced by electrical field stimulation) was also reduced by various measures employed to prevent PKC activity, as detailed below.

In primary cultures of rat SCG, the expression of PKC α, βI, βII, δ, ε and ζ has been documented by immunoblots, whereas PKC γ or μ was absent. All of the former isoforms, with one exception, PKC ζ, are downregulated by a long-lasting phorbol ester treatment [34]. Since exposure of the neurons to phorbol-12-myristate-13-acetate for 24 h did reduce oxotremorine M-induced noradrenaline release, a contribution of PKC ζ can be excluded. GF 109203 X is more potent at PKC α than at PKC β, and at least tenfold less potent at PKC δ and ε than at PKC β; atypical PKCs are not affected by GF 109203 X concentrations up to 1 μM. GÖ 6983, in contrast, is equipotent at virtually all PKC isoforms including the atypical ones, whereas Gö 6976 does not inhibit Ca^2+^-independent (δ and ε) and atypical PKC enzymes at concentrations up to 3 μM [30, 37]. In the present experiments (using concentrations of 0.01–1 μM), GÖ 6983 and GÖ 6976 turned out to be more potent in reducing oxotremorine M-induced noradrenaline release than GF 109203 X. Thus, the PKC enzymes involved can only be classical (Ca^2+^-sensitive) ones and include β rather than α subtypes. Hence, M_1_ receptors appear to engage PKC enzymes other than B_2_ bradykinin receptors to excite SCG neurons as the latter receptors were found to be linked to PKC δ and ε [34].

To elucidate the ionic mechanisms underlying the excitation of SCG neurons via M_1_ receptors, various types of experiments were performed. First, the intracellular concentrations of K^+^ and Cl^−^ were changed in perforated patch experiments, and only the changes in Cl^−^ caused alterations in oxotremorine M-induced depolarisations. Second, changes in membrane potentials caused by the mAChR agonist were compared with those caused by GABA, and both turned out to be depolarising. Moreover, both agonists triggered noradrenaline release in SCG cultures. Third, extracellular Cl^−^ was partially substituted by gluconate, and this enhanced noradrenaline release elicited by oxotremorine M and GABA, respectively, but not that induced by electrical field stimulation. Together, these results hint to an induction of a Cl^−^ conductance as one mechanism involved in the excitatory actions of M_1_ receptor activation.

This conclusion leads to at least two additional questions: (1) Why is the activation of a Cl^−^ conductance depolarising? (2) What are the channels mediating this Cl^−^ conductance? As far as question 1 is concerned, GABA has been reported previously to depolarise rat SCG neurons [1]. This depolarising action of GABA is based on the fact that these neurons accumulate high intracellular Cl^−^ concentrations of about 30 mM [3]. High intracellular Cl^−^ levels in neurons involve Cl^−^ uptake via Na^+^/K^+^/Cl^−^ co-transporters which can be blocked by bumetanide and related drugs [19]. The relevance of this mechanism for the excitatory actions of M_1_ receptors in SCG neurons was documented by the inhibition of oxotremorine M-evoked noradrenaline release by bumetanide and furosemide.

With respect to the Cl^−^ channels involved in the action of M_1_ receptors, several blockers have been employed in the present experiments. The non-selective Cl^−^ channel blockers niflumic acid and SITS reduced depolarisations as well as noradrenaline release triggered by oxotremorine M. This effect was specific for the depolarising action of the muscarinic agonist, as neither the oxotremorine M-dependent inhibition of K_v_7 channels nor electrically evoked noradrenaline release was altered by these agents. Whilst these results confirmed the contribution of some Cl^−^ channels to the excitatory action of M_1_ receptor activation, it remained unclear which of the numerous Cl^−^ channel subtypes might be involved [12, 32]. Previously, oxotremorine M had been reported to enhance a depolarisation-evoked Ca^2+^-dependent Cl^−^ current in rat SCG neurons [29]. More recently, mAChR agonists have been found to increase Ca^2+^-dependent Cl^−^ currents in interstitial cells of Cajal [39]. There is quite a variety of different Ca^2+^-activated Cl^−^ channels expressed in various cell types including neurons. Recently, TMEM16 proteins, in particular TMEM16A, also known as anoctamin 1 (ANO1), were found to contribute to the formation of Ca^2+^-activated Cl^−^ channels [4, 16]. Potent blockers that are selective for Ca^2+^-activated Cl^−^ channels in general and for TMEM16A in particular have been synthesized recently [31]. In the present experiments, these blockers (CaCCinh-A01 and T16Ainh-A01) reduced inward currents evoked by oxotremorine M and largely attenuated noradrenaline release triggered by the muscarinic agonist. Thus, TMEM16A is the most likely candidate to mediate the excitatory action of M_1_ receptor activation in SCG neurons. For comparison, sensory neurons were among the first cells that were revealed to exhibit Ca^2+^-dependent Cl^−^ currents and to express TMEM16A/ANO1 [16], and the latter channels have been demonstrated recently to be involved in the nociceptive activity of bradykinin [25]. TMEM16A/ANO1 is activated by Ca^2+^ concentrations in the low micromolar range [16], but oxotremorine M-induced increases in intracellular Ca^2+^ in SCG neurons as quantified by fura-2 microfluorometry do not exceed 1 μM and are only observed at depolarised membrane potentials [10]. However, the activation of TMEM16A/ANO1 in sensory neurons via G protein-coupled receptors relies on spatially restricted Ca^2+^ signals that hardly correlate with global cellular Ca^2+^, as determined with Ca^2+^ indicator microfluorometry [17]. Hence, the specific features of the Ca^2+^ signals that may link M_1_ receptors to TMEM16A/ANO1 remain to be determined.

Considering that the M_1_ receptor agonist led to an activation of PKC, on one hand, and to the gating of TMEM16A/ANO1 channels, on the other hand, the causal relation between these two events remained to be determined. The TMEM16A/ANO1 amino acid sequences in mammals contain putative phosphorylation sites for PKC [16], and currents through TMEM16A/ANO1 in biliary epithelial cells were found to be enhanced through an activation of classical PKC enzymes via P2Y receptors [13]. In the present experiments, PKC inhibitors attenuated the oxotremorine M-evoked currents that were otherwise reduced by blockers of Ca^2+^-dependent Cl^−^ currents and TMEM16A/ANO1 channels. Previously, the facilitation of depolarisation-evoked Ca^2+^-dependent Cl^−^ currents in SCG neurons by oxotremorine M was also reported to involve PKC [29]. Thus, PKC is involved in the activation of Ca^2+^-activated Cl^−^ channels via M_1_ receptors.

In summary, this report demonstrates that slow cholinergic excitation of sympathetic neurons involves mechanisms other than the inhibition of K_v_7 channels, which include an activation of classical PKCs and of Ca^2+^-activated Cl^−^ channels.

## Abbreviations

CaCCinh-A01: 6-(1,1-Dimethylethyl)-2-[(2-furanylcarbonyl)amino]-4,5,6,7-tetrahydrobenzo[*b*]thiophene-3-carboxylic acid
GÖ 6976: 12-(2-Cyanoethyl)-6,7,12,13-tetrahydro-13-methyl-5-oxo-5H-indolo[2,3-a]pyrrolo[3,4-c]carbazole
GÖ 6983: 3-[1-[3-(Dimethylamino)propyl]-5-methoxy-1H-indol-3-yl]-4-(1H-indol-3-yl)-1H-pyrrole-2,5-dione
mAChRs: Muscarinic acetylcholine receptors
nAChRs: Nicotinic acetylcholine receptors
PKC: Protein kinase C
SCG: Superior cervical ganglion
SITS: Disodium 4-acetamido-4′-isothiocyanato-stilbene-2,2′-disulfonic acid
T16Ainh-A01: 2-[(5-Ethyl-1,6-dihydro-4-methyl-6-oxo-2-pyrimidinyl)thio]-*N*-[4-(4-methoxyphenyl)-2-thiazolyl]acetamide
XE 991: 10,10-bis(4-Pyridinylmethyl)-9(10H)-anthracenone

## References

[1] Adams PR, Brown DA (1975). Actions of gamma-aminobutyric acid on sympathetic ganglion cells. J Physiol.

[2] Akaike N (1996). Gramicidin perforated patch recording and intracellular chloride activity in excitable cells. Prog Biophys Mol Biol.

[3] Ballanyi K, Grafe P (1985). An intracellular analysis of gamma-aminobutyric-acid-associated ion movements in rat sympathetic neurones. J Physiol.

[4] Berg J, Yang H, Jan LY (2012). Ca^2+^-activated Cl^−^ channels at a glance. J Cell Sci.

[5] Bernheim L, Mathie A, Hille B (1992). Characterization of muscarinic receptor subtypes inhibiting Ca^2+^ current and M current in rat sympathetic neurons. Proc Natl Acad Sci U S A.

[6] Boehm S (1998). Selective inhibition of M-type potassium channels in rat sympathetic neurons by uridine nucleotide preferring receptors. Br J Pharmacol.

[7] Boehm S, Huck S (1997). Noradrenaline release from rat sympathetic neurones triggered by activation of B2 bradykinin receptors. Br J Pharmacol.

[8] Brown AM (1967). Cardiac sympathetic adrenergic pathways in which synaptic transmission is blocked by atropine sulfate. J Physiol.

[9] Brown DA (1983). Slow cholinergic excitation—a mechanism for increasing neuronal excitability. Trends Neurosci.

[10] Del Río E, Bevilacqua JA, Marsh SJ, Halley P, Caulfield MP (1999). Muscarinic M_1_ receptors activate phosphoinositide turnover and Ca^2+^ mobilisation in rat sympathetic neurones, but this signalling pathway does not mediate M-current inhibition. J Physiol.

[11] Delmas P, Brown DA (2005). Pathways modulating neural KCNQ/M (K_v_7) potassium channels. Nat Rev Neurosci.

[12] Duran C, Thompson CH, Xiao Q, Hartzell HC (2010). Chloride channels: often enigmatic, rarely predictable. Annu Rev Physiol.

[13] Dutta AK, Woo K, Khimji A, Kresge C, Feranchak AP (2013). Mechanosensitive Cl^−^ secretion in biliary epithelium mediated through TMEM16A. Am J Physiol Gastrointest Liver Physiol.

[14] Hernandez CC, Zaika O, Tolstykh GP, Shapiro MS (2008). Regulation of neural KCNQ channels: signalling pathways, structural motifs and functional implications. J Physiol.

[15] Hoshi N, Zhang J-S, Omaki M, Takeuchi T, Yokoyama S, Wanaverbecq N, Langeberg LK, Yoneda Y, Scott JD, Brown DA, Higashida H (2003). AKAP150 signaling complex promotes suppression of the M-current by muscarinic agonists. Nat Neurosci.

[16] Huang F, Wong X, Jan LY (2012). International Union of Basic and Clinical Pharmacology. LXXXV: calcium-activated chloride channels. Pharmacol Rev.

[17] Jin X, Shah S, Liu Y, Zhang H, Lees M, Fu Z, Lippiat JD, Beech DJ, Sivaprasadarao A, Baldwin SA, Zhang H, Gamper N (2013). Activation of the Cl^−^ channel ANO1 by localized calcium signals in nociceptive sensory neurons requires coupling with the IP3 receptor. Sci Signal.

[18] Jones S, Brown DA, Milligan G, Willer E, Buckley NJ, Caulfield MP (1995). Bradykinin excites rat sympathetic neurons by inhibition of M current through a mechanism involving B2 receptors and G alpha q/11. Neuron.

[19] Kahle KT, Staley KJ, Nahed BV, Gamba G, Hebert SC, Lifton RP, Mount DB (2008). Roles of the cation-chloride cotransporters in neurological disease. Nat Clin Pract Neurol.

[20] Kristufek D, Koth G, Motejlek A, Schwarz K, Huck S, Boehm S (1999). Modulation of spontaneous and stimulation-evoked transmitter release from rat sympathetic neurons by the cognition enhancer linopirdine: insights into its mechanisms of action. J Neurochem.

[21] Kubista H, Kosenburger K, Mahlknecht P, Drobny H, Boehm S (2009). Inhibition of transmitter release from rat sympathetic neurons via presynaptic M(1) muscarinic acetylcholine receptors. Br J Pharmacol.

[22] Lechner SG, Hussl S, Schicker KW, Drobny H, Boehm S (2005). Presynaptic inhibition via a phospholipase C^−^ and phosphatidylinositol bisphosphate-dependent regulation of neuronal Ca^2+^ channels. Mol Pharmacol.

[23] Lechner SG, Mayer M, Boehm S (2003). Activation of M_1_ muscarinic receptors triggers transmitter release from rat sympathetic neurons through an inhibition of M-type K^+^ channels. J Physiol.

[24] Lee S-Y, Choi H-K, Kim S-T, Chung S, Park MK, Cho J-H, Ho W-K, Cho H (2010). Cholesterol inhibits M-type K^+^ channels via protein kinase C-dependent phosphorylation in sympathetic neurons. J Biol Chem.

[25] Liu B, Linley JE, Du X, Zhang X, Ooi L, Zhang H, Gamper N (2010). The acute nociceptive signals induced by bradykinin in rat sensory neurons are mediated by inhibition of M-type K^+^ channels and activation of Ca^2+^-activated Cl^−^ channels. J Clin Invest.

[26] Malhotra RK, Bhave SV, Wakade TD, Bhave AS, Wakade AR (1990). Effects of neurotransmitters and peptides on phospholipid hydrolysis in sympathetic and sensory neurons. FASEB J.

[27] Marrion NV (1997). Control of M-current. Annu Rev Physiol.

[28] Marrion NV, Smart TG, Marsh SJ, Brown DA (1989). Muscarinic suppression of the M-current in the rat sympathetic ganglion is mediated by receptors of the M_1_-subtype. Br J Pharmacol.

[29] Marsh SJ, Trouslard J, Leaney JL, Brown DA (1995). Synergistic regulation of a neuronal chloride current by intracellular calcium and muscarinic receptor activation: a role for protein kinase C. Neuron.

[30] Martiny-Baron G, Kazanietz MG, Mischak H, Blumberg PM, Kochs G, Hug H, Marme D, Schachtele C (1993). Selective inhibition of protein kinase C isozymes by the indolocarbazole Go 6976. J Biol Chem.

[31] Namkung W, Phuan P-W, Verkman AS (2011). TMEM16A inhibitors reveal TMEM16A as a minor component of calcium-activated chloride channel conductance in airway and intestinal epithelial cells. J Biol Chem.

[32] Nilius B, Droogmans G (2003). Amazing chloride channels: an overview. Acta Physiol Scand.

[33] Piper AS, Greenwood IA, Large WA (2002). Dual effect of blocking agents on Ca^2+^-activated Cl(−) currents in rabbit pulmonary artery smooth muscle cells. J Physiol.

[34] Scholze T, Moskvina E, Mayer M, Just H, Kubista H, Boehm S (2002). Sympathoexcitation by bradykinin involves Ca^2+^-independent protein kinase C. J Neurosci.

[35] Schwartz DD, Malik KU (1993). Cyclic AMP modulates but does not mediate the inhibition of [^3^H]norepinephrine release by activation of alpha-2 adrenergic receptors in cultured rat ganglion cells. Neuroscience.

[36] Trendelenburg U (1966). Observations on the ganglion-stimulating action of angiotensin and bradykinin. J Pharmacol Exp Ther.

[37] Way KJ, Chou E, King GL (2000). Identification of PKC-isoform-specific biological actions using pharmacological approaches. Trends Pharmacol Sci.

[38] Wess J, Eglen RM, Gautam D (2007). Muscarinic acetylcholine receptors: mutant mice provide new insights for drug development. Nat Rev Drug Discov.

[39] Zhu MH, Sung IK, Zheng H, Sung TS, Britton FC, O’Driscoll K, Koh SD, Sanders KM (2011). Muscarinic activation of Ca^2+^-activated Cl^−^ current in interstitial cells of Cajal. J Physiol.

